# An Analysis of Five *TrkB* Gene Polymorphisms in Schizophrenia and the Interaction of Its Haplotype with rs6265 BDNF Gene Polymorphism

**DOI:** 10.1155/2020/4789806

**Published:** 2020-04-13

**Authors:** Renata Suchanek-Raif, Paweł Raif, Małgorzata Kowalczyk, Monika Paul-Samojedny, Aleksandra Zielińska, Krzysztof Kucia, Wojciech Merk, Jan Kowalski

**Affiliations:** ^1^School of Pharmacy with the Division of Laboratory Medicine in Sosnowiec, Medical University of Silesia, Katowice, Poland; ^2^Department of Medical Genetics, Jedności 8 Street, 41-200 Sosnowiec, Poland; ^3^Department of Biosensors and Biomedical Signals Processing, Silesian University of Technology, Faculty of Biomedical Engineering, Roosevelta 40 Street, Zabrze, Poland; ^4^School of Medicine in Katowice, Medical University of Silesia, Katowice, Poland; ^5^Department of Psychiatry and Psychotherapy, Ziołowa 45 Street, 40-635 Katowice, Poland

## Abstract

**Aim:**

The BDNF dysfunction in the schizophrenia has been soundly documented. The *TrkB* gene is a high-affinity receptor of the BDNF that is changed in schizophrenia and mood disorders. The study had two aims: first, to identify whether the five nucleotide polymorphisms (SNPs) in *TrkB* gene are associated with a diagnosis of schizophrenia; and the latter, if any association exists between the *TrkB* SNPs and psychopathology, suicide attempts, and family history of schizophrenia in a Caucasian population.

**Methods:**

Case-control study (401 patients and 657 healthy controls) was used to examine a predisposition for schizophrenia. The tests for psychopathology, suicide attempts, and family history of schizophrenia were conducted only in patient group. The severity of the schizophrenia was measured using the five-factor model of the PANSS. In addition, the haplotype analysis for both the separate for SNPs of *TrkB* gene and in combination with the rs6265 SNP *BDNF* gene was conducted.

**Results:**

Our case-control study revealed that the genetic variants of rs10868235 (T/T polymorphic genotype) and rs1387923 (G/G polymorphic genotype) of the *TrkB* gene were associated with a higher risk of developing schizophrenia in men. However, the A/A wild genotype of rs1387923 was connected with a lower risk for both the development of and the family manifestation of schizophrenia in men. The G polymorphic allele of rs1565445 was associated with an increased risk of suicide in schizophrenia. The tested SNPs of the *TrkB* gene did not modulate the psychopathology of schizophrenia. The haplotype that was built with five SNPs in the *TrkB* gene was protective for men, but after joining the rs6265 SNP of the *BDNF* gene, a haplotype that was protective for women was created.

## 1. Introduction

Schizophrenia is a severe, chronic mental disease with an etiology that is still not fully understood. One of many hypotheses that have been put forward to explain its development involves a disturbance of the neurotrophins [1]. The TrkB (tropomyosin-related kinase B) receptor is involved in several important biological processes in neural tissues. This protein can promote both the prosurvival and the proapoptotic effects in response to neurotrophins. The TrkB receptor plays a critical role in signaling for the brain-derived neurotrophic factor (BDNF), which is one of the most important factors that play important roles in the survival and development of neurons and the establishment and maintenance of the synapses both during the development of the brain and in the brain of adults [2].

A BDNF dysfunction in schizophrenia either at a protein or an mRNA level has been strongly documented. The BDNF levels were lower in patients with schizophrenia in both the serum [1] and in the brain [3]. The levels of BDNF has also been correlated with the intensity of the psychopathological symptoms in patients with schizophrenia [4, 5]. The evidence has also revealed that the receptor TrkB levels are changed in schizophrenia patients. While the full-length TrkB levels were decreased, two truncated TrkB isoforms (trkB.t1 and trkB.shc) were increased in the prefrontal cortex [6, 7] and hippocampus in patients with schizophrenia [8].

The TrkB receptor has also been implicated in the differentiation and proliferation of neural cells. It promotes the neurite development and the synaptic plasticity in neurons. Moreover, TrkB also plays a role in neurotrophin-dependent calcium signaling in the glial cells [2]. TrkB exists in both the full-length and four C-terminal truncated isoforms (trkB.t1, trkB.shc, and trkB-T-TK without exon 23 or exon 24). In the brain, the full-length TrkB and trkB.t1 are the most abundant trkB isoforms of a receptor [9]. TrkB may also regulate the neurotransmitters. It has been revealed that this receptor might prompt the synthesis of gamma amino butyric acid (GABA) and the expression of the glutamate receptors. Additionally, schizophrenia is connected with a dysfunction of the GABA neurotransmission [10].

Studies on the significance of *TrkB* gene polymorphisms in schizophrenia have only been performed for the Chinese and Japanese populations [11, 12]. To the best of our knowledge, this is the first work that investigates the association between the *TrkB* gene polymorphisms and schizophrenia in Caucasian individuals. Data showed that the SNPs of the *TrkB* gene that were analysed for this paper have been associated with depression [13], suicides in individuals with a psychiatric disorder [14], the response to antidepressant treatment [15], and epilepsy [16].

The aim of the presented work was to evaluate the potential association between five single nucleotide polymorphisms (SNPs) (rs1867283, rs10868235, rs1565445, rs1387923, and rs2769605) of the *TrkB* gene and schizophrenia. We analysed the distribution of the genotypes, alleles, and haplotypes between patients with schizophrenia and healthy controls. Moreover, we assessed the associations the analysed SNPs with the clinical course of schizophrenia, as well as the family history of schizophrenia and suicide attempts. We also estimated any interaction with the polymorphic variants of the rs6265 *BDNF* gene *via* a haplotype analysis.

## 2. Methods

### 2.1. Subjects

The research group (*n* = 1058) included 401 patients and 657 healthy controls with Caucasian Polish origin. The patients with schizophrenia were being treated at the Clinic of Psychiatry in Katowice. The diagnosis was assigned by two independent psychiatrists based on the Diagnostic and Statistical Manual of Mental Disorders, 4th Edition, Text Revision (DSM-IV-TR) criteria. The Positive and Negative Syndrome Scale (PANSS) was used to evaluate the severity of the psychotic symptoms. The five-factor (positive (POS), negative (NEG), excitation (EXC), disorganization (DIS), and emotional distress (EMO) structure of the PANSS was applied. The first appearance of psychotic symptoms was considered the age of onset. The patients with the presence of depressive episodes, endocrine and autoimmune diseases, or comorbid mental disorders like anxiety disorder, schizoaffective disorder, organic brain disease, or substance dependence were excluded with research group. The anonymity of the patients was preserved. All of the patients were capable of understanding the study and provided written consent. The 76 patients had attempted suicide. In all cases, suicide attempts were confirmed by family members or significant others and by medical records. The data on family burden were obtained from an interview with family and by medical records. The 98 patients had the first degree relatives with schizophrenia.

The control group was consisted of unrelated healthy individuals. The persons with the current psychiatric problems, any other neurological disorders, and a family history of schizophrenia (verified during the interview) and chronic and acute physical illness such as an infection, autoimmune, or allergic diseases were not included in the group. The study was approved by the Bioethics Committee of Medical University of Silesia: KNW/0022/KB1/34/14.

### 2.2. Genotyping

Genomic DNA was isolated from peripheral blood using QIAampDNA Blood Mini Kit (Qiagen, Valencia, CA). The SNPs were genotyped using the TaqMan 5′-exonuclease allelic discrimination assay; the assay ID (Thermo Fisher Scientific Inc) of each SNP was C_1231356_20 for rs1867283, C_11923615_10 for rs10868235, C_1935385_10 for rs1565445, C_7423708_10 for rs1387923, C_16070662_10 for rs2769605, and C_11592758_10 for rs6265.

### 2.3. Statistical Analysis

Descriptive variables are presented as mean ± standard deviation (SD); qualitative data are expressed as percentage values. Differences in the allele, genotype, and haplotype frequencies were compared by the *χ*^2^ test. The two-way ANOVA and Tukey's HSD (honest significance test) post hoc test were used for comparisons of PANSS subscales and the age of onset. Homogeneity of variance was assessed by the Levene test. The extent of linkage disequilibrium (LD) expressed in terms of D′ and *r* coefficients; haplotypes frequencies and possible departure from the Hardy-Weinberg equilibrium (HWE) were estimated using the SNPStats software. Statistical calculations were performed by using the STATISTICA version 10 (http://www.statsoft.com) and SNPStats (http://bioinfo.inconcologia.net) and R-Studio. All results with *p* < 0.05 were accepted as statistically significant.

## 3. Results

### 3.1. Sample Characteristics

One thousand fifty-eight (*n* = 1058) subjects were enrolled in this study. Among the 401 patients, 159 (40%) were women, and 242 (60%) were men; the mean age ± standard deviation (SD) was 41.3 ± 12.3 years. The mean ± SD of the PANSS scores for the positive, negative, disorganization, emotional distress, and excitement subscales was 23.1 ± 5.4, 23.2 ± 5.8, 31.7 ± 6.9, 22.1 ± 4.9, and 20.5 ± 5.6, respectively. The mean age of the onset of the disease was 25.6 ± 6.8 years. The control group consisted of 657 unrelated subjects and included 315 (48%) women and 342 (52%) men. The mean age ± SD was 40.8 ± 8.6 years.

### 3.2. Genotypes and Alleles between Patients and Controls

The allele distribution did not significantly departure Hardy–Weinberg Equilibrium (HWE) neither among patients with schizophrenia (rs1867283, *p* = 0.11; rs10868235, *p* = 0.76; rs1565445, *p* = 0.9; rs1387923, *p* = 0.27; rs2769605, *p* = 0.13) nor for controls (rs1867283, *p* = 0.14; rs10868235, *p* = 0.7; rs1565445, *p* = 0.16; rs1387923, *p* = 0.16; rs2769605, *p* = 0.81).  Table 1 shows the frequencies of the genotypes and alleles of the five SNPs of the *TrkB* gene among the patients and the controls. There were no statistically significant differences in the distribution of genotypes and alleles for rs1867283, rs1565445, rs2769605, and rs10868235 SNPs either for entire group or the ones stratified by sex (Table 1).

The statistically significant differences in frequency genotypes were showed for rs1387923 (Table 1). The multiple logistic regression model showed that the A/A genotype (wild) of rs1387923 SNP was associated with a lower risk of schizophrenia in codominant and recessive genetic models (Table 2). The interaction analysis with a covariate sex showed that the A/A genotype (wild) had a lower risk than G/G genotype (polymorphic) in men but not women group. Moreover, the G/G (polymorphic) and G/A (heterozygous) genotypes in men had a high a risk of schizophrenia compared to women with the G/G (polymorphic) genotype (Table 3).

There were no statistically significant differences in the frequencies of either the genotypes or alleles between the patients and the controls for rs10868235 SNP used test *χ*^2^ (Table 1). However, the multiple logistic regression model adjusted for covariate sex showed that the T/T (polymorphic) and T/C (heterozygous) genotypes were associated with a high risk of schizophrenia in men compared to the T/T (polymorphic) genotype in women (Table 3).

### 3.3. PANSS Subscales

We performed the ANOVA test in order to examine the impact of the genotypes of analysed SNPs *TrkB* gene on the psychopathology, which was measured using the five-factor model of the PANSS scale. There were no statistical differences between the POS, NEG, DIS, EXC, and EMO subscales and any of the SNPs that were analysed (*p* > 0.05, data not shown).

### 3.4. Genotypes and Alleles among Men and Women with Schizophrenia

The group of men and women with schizophrenia did not show any significant differences in distribution either of the genotypes and alleles for any of the SNPs that were analysed (data no showed).

### 3.5. Suicide Attempts

Among the 401 patients, 76 (19%) had attempted suicide; mean age ± SD: 38 ± 10.9 years. The mean age of disease onset was 21.1 ± 5.8 years. There were 47 (62%) men (mean age: 36.8 ± 10.4 years) and 29 (38%) women (mean age: 41.4 ± 11.4 years) in the group.

The G polymorphic allele of rs1565445 SNP was a statistically significantly more frequent in group patients with a suicide attempts than patients without suicide attempts (35% vs. 26%, *χ*^2^ = 4, 7; *p* < 0.05). It is worth noting, the G/G genotype (polymorphic) rs1565445 SNP showed a statistically tendency to be more frequent in patients with suicide attempts than the patients without ones (12% vs. 7%, *χ*^2^ = 5.2, *p* = 0.07). The multiple logistic regression model showed that the combinational genotype G/G-G/A was associated with an increased risk for a suicide compared to the A/A wild genotype (the dominant model) (OR = 1.74, CI 95% (1.05-2.89); *p* < 0.05).

### 3.6. Family History of Schizophrenia

There were 98 patients with a family history of schizophrenia; mean age: 41.3 ± 12.1 years. The mean age of onset was 21.1 ± 6.3 years. There were 61 (53%) men (mean age: 37.0 ± 11.5 years) and 37 (47%) women (mean age: 48.0 ± 9.7 years) in the group. We found that the A/A genotype (wild) of rs1387923 SNP was more frequent in male patients without relatives with schizophrenia than male patients with family history of schizophrenia. We did not observe any significant differences in genotypes and allele distributions for rs1387923 SNP in women patients.

### 3.7. Haplotype Analysis

The haplotypes were formed by rs1867283 (G/A), rs10868235 (T/C), rs1565445 (A/G), rs1387923 (G/A), and rs2769605 (T/C). We found that the ATAAT haplotype was more frequent in control than in schizophrenia group (6% vs. 3%, respectively), and it was associated with a lower risk of schizophrenia, as well (OR = 0.44, 95% CI: 0.22-0.88, *p* < 0.05). After stratifying the groups by sex, this haplotype was protective only for men but not for women compared to the most frequent haplotype ATAGT (OR = 0.23, 95% CI: 0.08-0.7, *p* < 0.05).

We made the haplotype analysis between the five SNPs in *TrkB* gene and rs6265 SNP of *BDNF* gene. The haplotypes were formed by rs1867283 (G/A), rs10868235 (C/T), rs1565445 (A/G), rs1387923 (A/G), rs2769605 (C/T), and rs6265 (G/A). We found that two haplotypes were associated with a low risk of schizophrenia: the ATAATG haplotype (OR = 0.30; 95% CI: 0.11-0.78) and the GTAGCG haplotype (OR = 0.31; 95% CI: 0.10-0.89). The both haplotypes were more frequent in control than in schizophrenia group (5.5% vs. 1.5% and 3.4% vs. 1.3%, respectively). After stratifying the groups by sex, the GTAGCG haplotype was protective only for women but not for men, compared to the most frequent ATAGTG haplotype (OR = 0.10; 95% CI: 0.05-0.91, p < 0.05).

## 4. Discussion

The neurodevelopment theory is one of the ways through which the causes of schizophrenia were found. Neurotrophins, especially BDNF, are strong candidates. Data showed that not only the BDNF but also its receptor, TrkB, are changed in patients with schizophrenia [1, 17]. We conducted a case-control study of five SNPs in the *TrkB* gene in a Caucasian population. Our results show that the rs1387923 and rs10868235 SNPs are associated with a risk of schizophrenia. The G/G polymorphic genotype of rs1387923 and the T/T polymorphic genotype of rs10868235 had a higher risk of schizophrenia in men compared to women. Additionally, the A/A wild genotype of rs1387923 was found to be protective for men in comparison with men with the G/G polymorphic genotype.

There have been no papers that have analysed the SNPs of the *TrkB* gene in schizophrenia patients for the Caucasian population. A case-control study of Chinese population did not reveal any differences in the distribution of the alleles and genotypes for the rs1565445, rs1387923, and rs2769605 SNPs of the *TrkB* gene in schizophrenia [12]. A study of twelve *TrkB* gene SNPs of the *TrkB* gene for a Japanese population also did not reveal any association [11]. It worth mentioning that the T/T polymorphic genotype rs10868235 of the *TrkB* gene, which in our study was associated with a higher risk of schizophrenia, has also been connected with other diseases of central nervous system such as epilepsy. The T/T genotype showed a statistical trend for increase in patients with epilepsy [16]. Moreover, an association between rs10868235 was observed in the impulsivity phenotypes as well as in quantitative hopelessness among healthy adolescents [18]. Data revealed that the SNPs of the *TrkB* gene affected the mechanisms that are used to cope with stress in young Japanese people. It is worth stressing that individuals with the T/T genotype (polymorphic) of rs10868235 had lower scores for the cognitive strategies for relieving stress [19]. Cognitive impairment is common in schizophrenia patients, and stress is an important factor that modulates both the occurrence of and the course of schizophrenia [20, 21].

We analysed five SNPs in the *TrkB* gene and investigated the psychopathology of schizophrenia. We used the five-factor PANSS model, which was developed by van der Gaag et al. [22]. This model is more stable than other published models and reflects the clinical reality more accurately [22]. Our results showed that the analysed SNPs of the *TrkB* gene are not connected with the intensity of schizophrenia as measured with the five-factor PANSS scale. To the best of our knowledge, there are no papers that have assessed this type of interaction. A link between the psychopathology of schizophrenia and the TrkB receptor may be supported by the following findings. The SNPs of the *TrkB* gene were associated with the emotional arousal or the impulsivity phenotypes in healthy people [18, 23]. It was found that the BDNF-TrkB signaling affects the plasticity mechanisms at the GABA-ergic synapses [10]. In schizophrenia, several alterations were revealed in GABA neurotransmission. It was suggested that abnormalities in the GABA circuits might be a crucial factor in the pathophysiology of the cognitive dysfunction in patients with schizophrenia [3].

Suicide is a significant problem in schizophrenia. The risk of suicide is higher among schizophrenia patients [24]. We found that the rs1565445 of the *TrkB* gene is associated with suicide attempts. The G allele and G/G genotype (polymorphic) were more frequent among patients who attempted suicide and were connected with higher risk of suicide (OR = 1.74). There were no data for an association between rs1565445 or the other SNPs of the *TrkB* gene and suicide in schizophrenia. Bremer et al. [25] analysed 25 SNPs of the *TrkB* gene and found that both the rs1565445 and rs1387923 SNPs of the *TrkB* gene were connected with the response to lithium in patients with bipolar disorder in the Caucasian population. A history of suicidal ideation or post-traumatic stress disorder decreased the treatment response for the genotypes either rs1565445 and rs1387923 polymorphisms [25]. Stress is one of the crucial elements that play a role in both the development of and in the psychopathology of schizophrenia [20]. Stress decreased the levels of neurotrophins, especially the BDNF levels, in the hippocampus, which is the area of brain that is seriously damaged in the course of schizophrenia [26]. Another study showed the role of the genotypes of rs1565445 and rs1387923 in treatment-resistant depression (TRD). The authors found that there were differences in the frequencies for either the alleles or genotypes of those SNPs in the TRD group compared to the non-TRD group. The haplotype T-T (built with rs1565445 and rs1387923 SNPs) was connected with a 1.41-fold increased risk of TRD [13]. The other SNPs of *TrkB* that were analysed in our paper were also evaluated for their role in suicidal behavioural and antidepressant treatment for major depression, bipolar I disorder, and in healthy adolescents. The rs10868235, rs2769605, rs1387923, and several other SNPs of the *TrkB* gene were associated with suicidal ideation in depression and affected the response to antidepressants [27, 28]. The rs1867283 was suggested to influence suicide attempts, the impulsivity phenotypes, and the loss of hope among adolescents in France [18]. As was mentioned above, the SNPs of the *TrkB* gene have been associated with the mechanisms that are used to cope with stress in young people [19].

Family and twin studies suggest that there is a higher risk of developing schizophrenia among the relatives of the affected person [29]. We showed that the A/A genotype (wild) of rs1387923 was higher in men with no family history of schizophrenia. To the best of our knowledge, there have been no papers that have assessed this kind of association. We would like to stress that in our study, this genotype (A/A genotype for rs1387923) was connected with a lower risk of developing schizophrenia compared to the G/G genotype in men.

Our haplotype analysis showed the sex differences in the risk of developing schizophrenia for the polymorphisms of the *TrkB* and *BDNF* genes. We found that the ATAAT haplotype (built with five SNPs in the *TrkB* gene) was associated with a lower risk of schizophrenia for men but that the GTAGCG haplotype (build with five of the analysed SNPs *TrkB* and rs6265 *BDNF* SNP) was connected with a lower risk of schizophrenia for women. The rs6265 BDNF SNP is the most intensively studied SNP of the *BDNF* gene. A meta-analysis showed that the rs6265 SNPs of the *BDNF* gene are not associated with schizophrenia in either Caucasian or Asian populations [30]. However, it was also revealed that there were sex differences in the impact of the rs6265 SNP *BDNF* gene on the cortisol level under acute stress. A higher level was found in the val/val of men but not women [31]. The importance of sex differences in the circuit of neurotransmitters on either a predisposition for or the intensity of psychiatric disorders has been proposed. Data showed the sex differences in the activation of the hypothalamic pituitary adrenal axis and in the release of the corticotrophin releasing factor (CRF), which is one of the factors that regulate cognitive changes due to stress [32, 33]. It worth stressing that while on the one hand, the BDNF is a trophic factor for the dopaminergic, GABAergic, serotoninergic, cholinergic, or noradrenergic neurons [34], on the other, the effect of the estrogens and the glucocorticoids on the *BDNF* gene expression has been documented [35, 36]. These studies suggest that the relationship between the BDNF and hormones may be mutually reciprocal. Therefore, the polymorphic changes in the genes that alter the structure of proteins (such as rs6265 in the BDNF gene) may not be unrelated to these relationships.

## 5. Conclusion

Our research revealed that the genetic variants of rs10868235 (T/T polymorphic genotype) and rs1387923 (G/G polymorphic genotype) *TrkB* gene were associated with a higher risk of developing schizophrenia in men. Moreover, the A/A wild genotype of rs1387923 was connected with a lower risk for both the development of and the family manifestation of schizophrenia in men. We did not observe any significant differences in the distribution of genotypes and alleles between the patients with schizophrenia and the controls for the rs1867283, rs1565445, and rs2769605 SNPs *TrkB* gene. The G allele and the G/G genotype (polymorphic) of rs1565445 were associated with an increased risk of suicide among patients. None of the tested SNPs of the *TrkB* gene modulated the psychopathology of schizophrenia. We found that the haplotype built with five SNPs in the *TrkB* gene was protective for men, but when we added the rs6265 SNP of *BDNF* gene, we observed a haplotype that was protective for women. Our findings are especially true for the paranoid subtype of schizophrenia with no coexisting depressive episodes.

### 5.1. Limitation

Further analyses are necessary in order to clarify the role of the analysed SNPs of the *TrkB* gene in the pathogenesis of schizophrenia. Our study requires replication in a larger group, especially for the analyses in the subgroups such as suicide attempts and a family history of schizophrenia, in order to possibly propose the polymorphism with more relevant results as a possible marker of predisposition to schizophrenia or its intensity of psychopathology.

## Figures and Tables

**Table 1:** Analysis of genotype and allele distributions five SNPs *TrkB* gene for the whole population and stratified by sex.

SNP	Genotype Allele	Control n (%)	Patients n (%)	*Χ* ^2^	*p*	Male		*Χ* ^2^	*p*	Female		*Χ* ^2^	*p*
						Control *n* (%)	Patients *n* (%)			Control *n* (%)	Patients *n* (%)		
rs1867283	G/G	180 (27)	98 (24)	2.44	0.29	100 (29)	56 (23)	3.65	0.16	80 (26)	42 (27)	0.23	0.88
	G/A	309 (47)	184 (46)			153 (45)	109 (45)			156 (49)	75 (47)		
	A/A	168 (26)	119 (30)			89 (26)	77 (32)			79 (25)	42 (26)		
	G	669 (51)	308 (47)	2.34	0.12	353 (52)	221 (46)	3.77	0.06	316 (49)	159 (50)	0.01	0.99
	A	645 (49)	422 (53)			331 (48)	263 (54)			314 (51)	159 (50)		

rs1565445	A/A	333 (51)	208 (52)	1.09	0.57	173 (50)	128 (53)	1.22	0.54	160 (51)	80 (50)	0.19	0.90
	A/G	259 (39)	161 (40)			133 (39)	96 (39)			126 (40)	66 (42)		
	G/G	65 (10)	32 (8)			36 (11)	19 (8)			29 (9)	13 (8)		
	A	925 (70)	577 (72)	0.50	0.47	479 (70)	351 (73)	0.73	039	446 (70)	226 (71)	0.01	0.99
	G	389 (30)	225 (28)			205 (30)	133 (27)			184 (30)	92 (29)		

rs2769605	C/C	119 (18)	86 (21)	2.28	0.31	64 (19)	49 (20)	0.21	0.89	55 (18)	37 (23)	3.23	0.19
	C/T	326 (50)	183 (46)			161 (47)	112 (46)			165 (52)	71 (45)		
	T/T	212 (32)	132 (33)			117 (34)	81 (34)			95 (30)	51 (32)		
	C	564 (43)	355 (44)	0.31	0.57	289 (42)	210 (43)	0.11	0.74	275 (44)	145 (46)	0.25	0.61
	T	750 (57)	447 (56)			395 (58)	274 (57)			355 (56)	173 (54)		

rs10868235	C/C	153 (23)	105 (26)	1.14	0.56	86 (25)	57 (24)	0.22	0.89	68 (22)	48 (30)	4.4	0.11
	C/T	334 (51)	197 (49)			173 (51)	122 (50)			161 (51)	75 (47)		
	T/T	170 (26)	99 (25)			84 (24)	63 (26)			86 (27)	36 (23)		
	C	640 (49)	407 (51)	0.75	0.38	343 (50)	236 (49)	0.16	0.68	297 (47)	171 (54)	3.5	0.06
	T	674 (51)	395 (49)			341 (49)	248 (51)			333 (52)	147 (46)		

rs1387923	A/A	173 (27)	76 (19)	7.56	<0.05	92 (27)	45 (19)	5.54	0.06	81 (26)	31 (20)	2.93	0.23
	A/G	310 (47)	210 (52)			159 (46)	122 (50)			151 (48)	88 (55)		
	G/G	174 (26)	115 (29)			91 (27)	75 (31)			83 (26)	40 (25)		
	A	656 (50)	362 (45)	4.38	<0.05	343 (50)	212 (44)	4.32	<0.05	313 (50)	150 (47)	0.43	0.51
	G	658 (50)	440 (55)			341 (50)	272 (56)			317 (50)	168 (53)		

**Table 2:** Association between rs1387923 *TrkB* gene and risk of schizophrenia under different genetic models.

Model	Genotype rs1387923	Control	Schizophrenia	OR (95% CI)	*p* value	AIC
Codominant	G/G	174 (27%)	115 (29%)	1.0	*p* < 0.05	1397.4
	G/A	310 (47%)	210 (52%)	1.04 (0.77-1.39)		
	A/A	173 (26%)	76 (19%)	**0.67 (0.47-0.96)**		

Recessive	G/G-G/A	484 (74%)	325 (81%)	1.0	*p* < 0.05	1395.5
	A/A	173 (26%)	76 (19%)	**0.65 (0.48-0.89)**		

Abbreviations: OR: odds ratio; CI: confidence interval.

**Table 3:** Interaction analysis between rs1387923 and rs10868235 *TrkB* gene and sex on the risk of schizophrenia.

Genotype	Sex	Control (*n*)	Schizophrenia (*n*)	OR (95% CI)	*p* value
rs1387923					
G/G	Female	83	40	**1.00**	
G/G	Male	91	75	1.71 (1.05-2.78)	*p* < 0.05
G/A	Male	159	122	1.59 (1.02-2.48)	*p* < 0.05
rs10868235					
T/T	Female	86	36	**1.00**	
T/T	Male	84	63	1.79 (1.08-2.98)	*p* < 0.05
C/T	Male	173	122	1.68 (1.07-2.65)	*p* < 0.05

## Data Availability

Answer: Yes. Comment: The data used to support the findings of this study are available from the corresponding author upon request.

## Supplementary Materials

Supplementary Materials Table 4: the results of ANOVA (sex and genotype distributions) for PANSS subscales schizophrenia. Abbreviations: F: Fisher test, *p* value. Table 5: genotype and allele distributions of five SNPs *TrkB* gene in women and men with schizophrenia. Table 6: genotype and allele distributions of five SNPs *TrkB* gene in schizophrenia patients with and without suicide attempts. Table 7: genotype and allele distributions of the rs1387923 *TrkB* gene among patients with and without family history of schizophrenia.
