# Supplementary material for: An Analysis of Five TrkB Gene Polymorphisms in Schizophrenia and the Interaction of Its Haplotype with rs6265 BDNF Gene Polymorphism
Source: Dis Markers. 2020 Apr 13;2020:4789806. doi: 10.1155/2020/4789806 (PMC7174942; doi:10.1155/2020/4789806)
Supplement: Supplementary Materials — Table 4: the results of ANOVA (sex and genotype distributions) for PANSS subscales schizophrenia. Abbreviations: F: Fisher test, p value. Table 5: genotype and allele distributions of five SNPs TrkB gene in women and men with schizophrenia. Table 6: genotype and allele distributions of five SNPs TrkB gene in schizophrenia patients with and without suicide attempts. Table 7: genotype and allele distributions of the rs1387923 TrkB gene among patients with and without family history of schizophrenia. [file 4789806.f1.docx]

**Supplementary tables**

Table 4. The results of ANOVA (sex and genotype distributions) for PANSS subscales schizophrenia. Abbreviations: F - Fisher test, p-value.

|  | **PANSS** | | **genotype** | | **sex** | | **sex × genotype** | |
| --- | --- | --- | --- | --- | --- | --- | --- | --- |
|  | **subscales** | | **F** | ***p*** | **F** | ***p*** | **F** | ***p*** |
| rs1867283 | positive | 0.12 | | 0.88 | 3.15 | 0.17 | 5.87 | 0.35 |
|  | negative | 1.01 | | 0.36 | 2.05 | 0.18 | 0.17 | 0.83 |
|  | disorganization | 0.05 | | 0.94 | 3.40 | 0.15 | 0.54 | 0.57 |
|  | emotional distress | 2.45 | | 0.13 | 3.50 | 0.10 | 2.85 | 0.10 |
|  | excitation | 1.86 | | 0.19 | 5.26 | 0.08 | 4.57 | 0.09 |
| rs1565445 | positive | | 1.33 | 0.26 | 3.07 | 0.09 | 0.54 | 0.58 |
|  | negative | | 0.88 | 0.41 | 2.10 | 0.14 | 0.14 | 0.87 |
|  | disorganization | | 1.30 | 0.27 | 3.41 | 0.07 | 0.19 | 0.82 |
|  | emotional distress | | 1.17 | 0.30 | 3.37 | 0.07 | 0.44 | 0.64 |
|  | excitation | | 3.21 | 0.04 | 6.03 | 0.01 | 1.28 | 0.27 |
| rs2769605 | positive | | 0.32 | 0.72 | 3.07 | 0.10 | 1.65 | 0.19 |
|  | negative | | 0.98 | 0.37 | 2.22 | 0.14 | 0.94 | 0.39 |
|  | disorganization | | 0.12 | 0.88 | 3.38 | 0.11 | 0.48 | 0.61 |
|  | emotional distress | | 0.45 | 0.63 | 3.25 | 0.11 | 1.06 | 0.34 |
|  | excitation | | 0.24 | 0.78 | 4.71 | 0.09 | 2.02 | 0.10 |
| rs10868235 | positive | | 0.93 | 0.39 | 2.93 | 0.09 | 0.93 | 0.39 |
|  | negative | | 1.31 | 0.26 | 1.93 | 0.16 | 0.11 | 0.89 |
|  | disorganization | | 0.59 | 0.55 | 3.57 | 0.59 | 1.17 | 0.33 |
|  | emotional distress | | 1.98 | 0.13 | 3.47 | 0.16 | 2.44 | 0.11 |
|  | excitation | | 2.30 | 0.10 | 5.69 | 0.17 | 1.64 | 0.19 |
| rs1387923 | positive | | 0.24 | 0.81 | 3.09 | 0.08 | 0.45 | 0.63 |
|  | negative | | 0.80 | 0.44 | 1.97 | 0.16 | 0.44 | 0.66 |
|  | disorganization | | 0.64 | 0.54 | 3.30 | 0.07 | 0.13 | 0.81 |
|  | emotional distress | | 0.07 | 0.93 | 3.236 | 0.08 | 0.30 | 0.73 |
|  | excitation | | 0.51 | 0.60 | 6.030 | 0.08 | 0.83 | 0.43 |

Table 5. Genotype and allele distributions of five SNPs *TrkB* gene in women and men with schizophrenia.

| **Polymorphisms** | N (%) | | | | |  | | | |
| --- | --- | --- | --- | --- | --- | --- | --- | --- | --- |
|  | **Genotype** | | | | | **Allele** | | | |
| rs1867283 | **G/G** | **G/A** | **A/A** | ***χ^2^*** | ***p*** | **G** | **A** | ***χ^2^*** | ***P*** |
| ***Women*** | 42 (26) | 75 (48) | 42 (26) | 1.45 | 0.48 | 159 (50) | 159 (50) | 1.28 | 0.25 |
| ***Men*** | 56 (23) | 109 (45) | 77 (32) |  |  | 221 (46) | 263 (56) |  |  |
| rs10868235 | **C/C** | **C/T** | **T/T** | ***χ^2^*** | ***p*** | **C** | **T** | ***χ^2^*** | ***p*** |
| ***Women*** | 48 (30) | 75 (47) | 36 (23) | 2.26 | 0.32 | 171 (54) | 147 (46) | 1.73 | 0.19 |
| ***Men*** | 57 (24) | 122  (50) | 63  (26) |  |  | 236 (49) | 248 (51) |  |  |
| rs1565445 | **A/A** | **A/G** | **G/G** | ***χ^2^*** | **p** | **A** | **G** | ***χ^2^*** | ***p*** |
| ***Women*** | 80 (50) | 66 (42) | 13 (8) | 0.25 | 0.87 | 226 (71) | 92 (29) | 0.13 | 0.71 |
| ***Men*** | 128 (53) | 95 (39) | 19 (8) |  |  | 351 (73) | 133 (27) |  |  |
| rs1387923 | **A/A** | **A/G** | **G/G** | ***χ^2^*** | ***p*** | **A** | **G** | ***χ^2^*** | ***p*** |
| ***Women*** | 31 (20) | 88 (55) | 40 (25) | 1.62 | 0.44 | 150 (47) | 168 (53) | 0.74 | 0.38 |
| ***Men*** | 45 (19) | 122 (50) | 75 (31) |  |  | 212 (44) | 272 (56) |  |  |
| rs2769605 | **C/C** | **C/T** | **T/T** | ***χ^2^*** | **p** | **C** | **T** | ***χ^2^*** | ***p*** |
| ***Women*** | 37 (23) | 71 (45) | 51 (32) | 0.52 | 0.77 | 145 (46) | 173 (54) | 0.29 | 0.58 |
| ***Men*** | 49 (20) | 112 (46) | 81 (34) |  |  | 210 (43) | 274 (57) |  |  |

Table 6. Genotype and allele distributions of five SNPs *TrkB* gene in schizophrenia patients with and without suicide attempts.

| **SNP** | N (%) | | | | |  | | | |
| --- | --- | --- | --- | --- | --- | --- | --- | --- | --- |
|  | **Genotype** | | | | | **Allele** | | | |
| rs1867283 | **G/G** | **G/A** | **A/A** | ***χ^2^*** | ***p*** | **G** | **A** | ***χ^2^*** | ***P*** |
| **with suicide attempts** | 21 (28) | 39 (51) | 16 (21) | *3.36* | *0.18* | 81 (53) | 71 (47) | *2.34* | *0.14* |
| **without suicide attempts** | 103 (24) | 145 (44) | 77 (32) |  |  | 299 (46) | 351 (54) |  |  |
| rs10868235 | **C/C** | **C/T** | **T/T** | ***χ^2^*** | ***p*** | **C** | **T** | ***χ^2^*** | ***p*** |
| **with suicide attempts** | 22 (29) | 36 (47) | 18 (24) | *0.37* | *0.83* | 80 (53) | 72 (47) | *0.18* | *0.67* |
| **without suicide attempts** | 83 (26) | 161 (50) | 81(25) |  |  | 327 (50) | 323 (50) |  |  |
| rs1565445 | **A/A** | **A/G** | **G/G** | ***χ^2^*** | **p** | **A** | **G** | ***χ^2^*** | ***p*** |
| **with suicide attempts** | 31 (41) | 36 (47) | 9 (12) | *5.18* | *0.07* | 98 (65) | 54 (35) | *4.74* | ***<0.05*** |
| **without suicide attempts** | 177 (554) | 125 (38) | 23 (7) |  |  | 479 (74) | 171 (26) |  |  |
| rs1387923 | **A/A** | **A/G** | **G/G** | ***χ^2^*** | ***p*** | **A** | **G** | ***χ^2^*** | ***p*** |
| **with suicide attempts** | 19 (25) | 41 (54) | 16 (21) | *3.75* | *0.15* | 79 (52) | 73 (48) | *3.20* | *0.07* |
| **without suicide attempts** | 57 (18) | 169 (52) | 99 (30) |  |  | 283 (44) | 367 (56) |  |  |
| rs2769605 | **C/C** | **C/T** | **T/T** | ***χ^2^*** | **p** | **C** | **T** | ***χ^2^*** | ***p*** |
| **with suicide attempts** | 19 (25) | 37 (47) | 20 (28) | *1.98* | *0.37* | 75 (49) | 77 (51) | *1.71* | *1.19* |
| **without suicide attempts** | 67 (21) | 146 (45) | 112 (34) |  |  | 280 (43) | 37 (57) |  |  |

Table 7. Genotype and allele distributions of the rs1387923 *TrkB* gene among patients with and without family history of schizophrenia.

| Genotype/  Allele  rs1387923 | Male patients  n (%) | |  |  | Female patients  n (%) | | |  | |  |
| --- | --- | --- | --- | --- | --- | --- | --- | --- | --- | --- |
|  | ***with***  ***family history*** | ***without family history*** | *Χ^2^* | *p* | ***with family history*** | ***without family history*** | *Χ^2^* | | *p* | |
| A/A | 7 (11) | 38 (21) | *6.25* | *<0.05* | 10 (27) | 21 (17) | *4.32* | | *0.11* | |
| A/G | 39 (64) | 83 (46) |  |  | 15 (41) | 73 (60) |  | |  | |
| G/G | 15 (25) | 60 (33) |  |  | 12 (32) | 28 (23) |  | |  | |
| A | 53 (43) | 159 (44) | *0.21* | *0.99* | 35 (47) | 115 (47) | *0.14* | | *0.99* | |
| G | 69 (57) | 203 (56) |  |  | 39 (53) | 129 (53) |  | |  | |

**List of tables**

Table 4. The results of ANOVA (sex and genotype distributions) for PANSS subscales schizophrenia. Abbreviations: F - Fisher test, p-value.

Table 5. Genotype and allele distributions of five SNPs *TrkB* gene in women and men with schizophrenia.

Table 6. Genotype and allele distributions of five SNPs *TrkB* gene in schizophrenia patients with and without suicide attempts.

Table 7. Genotype and allele distributions of the rs1387923 *TrkB* gene among patients with and without family history of schizophrenia.
